# Psychometric properties of the Persian short form of the Stigma of Suicide Scale

**DOI:** 10.3389/fpsyt.2024.1394237

**Published:** 2024-07-26

**Authors:** Alireza Jafari, Ali Mohammad Mokhtari, Mahdi Moshki, Fatemeh Rahmani, Fatemehzahra Naddafi, Mahbobeh Nejatian

**Affiliations:** ^1^ Department of Health Education and Health Promotion, School of Health, Social Development and Health Promotion Research Center, Gonabad University of Medical Sciences, Gonabad, Iran; ^2^ Department of Epidemiology and Biostatistics, School of Health, Social Development and Health Promotion Research Center, Gonabad University of Medical Sciences, Gonabad, Iran; ^3^ Department of Health Education and Health Promotion, School of Health, Social Determinants of Health Research Center, Gonabad University of Medical Sciences, Gonabad, Iran; ^4^ Student Research Committee, Gonabad University of Medical Sciences, Gonabad, Iran; ^5^ Social Determinants of Health Research Center, Gonabad University of Medical Sciences, Gonabad, Iran

**Keywords:** suicide, stigma, validity, translation, reliability

## Abstract

**Objective:**

Suicide stigma is a major obstacle to suicide prevention, resulting in a decrease in mental help seeking. This study aimed to survey the psychometric characteristics of the Persian short form of the Stigma of Suicide Scale (SOSS).

**Methods:**

This psychometric study was conducted on 956 people (EFA = 399 samples, CFA = 557) in 2022 to evaluate the validity (face, content, and structure validity) and reliability (Cronbach’s alpha coefficient, McDonald Omega coefficient, and intraclass correlation coefficient) of the SOSS. The structural validity of the scale was assessed by confirmatory factor analysis (CFA) and exploratory factor analysis (EFA).

**Results:**

The scores of S-CVI/Ave and CVR for SOSS were 0.982 and 0.921, respectively. In the EFA section, three factors with eigenvalues above one were shown, and 60.60% variance of the scale was explained by these factors, and one question was eliminated due the factor loading less than 0.4 and also moving to an irrelevant factor. Finally, based on the goodness-of-fit indices (such as RMSEA = .077, CFI= .902, IFI= .903, GFI= .915), the Persian short form of SOSS was approved with 15 items and three factors of Glorification/Normalization (4 items), Stigma (7 items), Isolation/Depression (4 items). The McDonald Omega coefficient, Cronbach’s alpha coefficient, and ICC for SOSS were 0.841, 0.834, and 0.881, respectively.

**Conclusion:**

In this study, the Persian short form of the SOSS was approved with 15 items and 3 factors, and this scale is an appropriate instrument for determining the status of suicide stigma among general population.

## Introduction

Suicide is a critical issue that kills approximately 800,000 people annually and accounts for 1.5% of global death. Although suicide attempt rates are about 20 to 30 times higher than complete suicide rates (1–3). Suicide can have a lot of costs on the health system and devastating effects on societies and families. Given the importance of suicide today, a priority of the World Health Organization is the reduction of suicide deaths (4, 5). However, suicide is not a simple phenomenon and is the result of the impact of various cultural, demographic, social, psychological, and environmental factors (6–8). Therefore, several such cases should be considered to prevent suicide (6–8).

The impact of social and cultural factors such as suicide stigma has been shown in numerous studies (3, 6, 9, 10). A review study found that suicide stigma exists in many societies (11) and according to the World Health Organization, suicide stigma is one of the biggest and main obstacles in preventing suicide (12). Stigma is the sign of shame, beliefs, evaluations, and negative attitudes that refer to a behavior or attribute (13, 14). Suicide stigma is defined as a negative attitude of individuals in the community toward those who committed suicide (15).

In general, stigma can have a variety of negative consequences on healthy behaviors such as hiding illness, seeking health services, using available resources, psychological responses, social relationships, and adherence to treatment (16–20). Suicide stigma can also specifically act as a barrier to reducing help-seeking and support and increasing the risk of suicide and psychological distress. Finally, suicide stigma will also reduce the desire of people to participate in suicide prevention interventions and programs and will be an essential obstacle to suicide prevention (19, 21).

In a study, Iranian women who had previously committed suicide mentioned that the thought of suicide and the desire to commit suicide had been hidden from others because of fears of stigmas such as mental illness, unacceptable behavior not being a religious person and illegitimate sex (22). Therefore, suicide stigma is one of the important factors that causes people who have suicide thoughts or suicide attempts to not desire to seek and receive mental health services (15, 23).

One of the appropriate tools for examining the status of suicide stigma is the suicide stigma scale (SOSS) that designed by Batterham et al., and contains 16 questions and three factors (24). This questionnaire has been translated and its validity and reliability have been examined in different languages and countries (3, 10, 25, 26). Due to the lack of a valid tool in the Iranian community and the need for a proper tool for examining the status of suicide stigma, the present study was conducted to evaluate the validity and reliability of SOSS in Iranian public population.

## Methods

This study was conducted among 956 public population in Gonabad (Iran) in 2022 to evaluate the psychometric properties of the Persian short form of the SOSS.

### Sample size

Different sources recommend that a sample size of more than 500 is suitable for performing factor analysis (27, 28). Exploratory factor analysis (EFA) and confirmatory factor analysis (CFA) should not be evaluated in the same data (29). In this study, due to the high risk of overfitting, EFA and CFA were conducted in different samples. EFA was conducted on 399 samples and CFA was conducted on 557 samples.

### Sampling method

The general population of Gonabad city (Iran) was recruited for the study by proportional stratified sampling. In Iran, all people are under the care of comprehensive community health centers (CCHC) and have an electronic health file. Therefore, first, all the CCHCs (n=3) located in different areas of Gonabad were considered as strata. In Iran, the demographic and health information of all people from birth to death is recorded in the Sib system, and each person has an electronic file. In the Sib system, the number and characteristics of all the people covered by each CCHC are completely and accurately determined. In this study, the Sib system was used as a framework for sampling. Initially, a raw population was determined for each CCHC. Then, after applying the inclusion criteria, i.e., age over 18 years and residence for more than one year in Gonabad, the target population was determined in each center and according to the population ratio of each center, the sample size of each center was determined. Finally, simple random sampling was performed according to the sample size allocated to each stratum. After selecting the samples and explaining the study procedure to the participants, they signed the informed consent form and completed the questionnaire by self-report.

### Instruments

Demographic section: In this part, demographic information was assessed.

Stigma of Suicide Scale (SOSS): The questionnaire was designed and evaluated by Batterham et al. (24). The short form of this tool consists of 16 questions extracted from the long form with 58 items. This short form of scale has three factors: glorification/normalization (4 items), stigma (8 items) and isolation/depression (4 items). The questions are measured on a five-point Likert scale (strongly disagree = 1, to, strongly agree = 5). The mean score in each subscale was calculated and the score range for each subscale is between 1–5, and higher score indicating higher glorification/normalization, stigma, and isolation/depression (24).

### Translation and cultural adaptation

In this study, the translation process was conducted after obtaining written permission and the original English version of the SOSS questionnaire from the developer.​​​​​​​ Then, based on the translation guideline (30), two translators independently translated the English version into Persian. Then, the two Persian versions were merged by the research team, and the differences were discussed. The merged Persian version was then translated into English by two translators who were blinded to the original version. Then the two English versions were merged by the research team and compared with the original SOSS version. Finally, the final merged English version of the SOSS was translated into Persian and used to examine its psychometric properties.

### Validity

After creating the final Persian version, the scale was sent to 8 specialists of Psychology and specialists of Health Education and Health Promotion and reviewed in terms of content validity (qualitative and quantitative methods) and face validity (qualitative method). Also, in qualitative face validity, the items of SOSS were assessed by 9 participants of the target group. In quantitative content validity, scale content validity index averaging (S-CVI/Ave) and content validity ratio (CVR) were assessed. In S-CVI/Ave, each item of SOSS was assessed in terms of relevance (31). The acceptable score for S-CVI/Ave is more than 0.9 (32) and the acceptable score for CVR is more than 0.75 (33).

### EFA

EFA was performed using SPSS software version 24. In this section, the number of extractable factors was examined. Therefore, eigenvalues more than one, factor loading above 0.4, and a maximum of 25 rotation repetitions (Extraction Method: Maximum Likelihood, varimax rotation) were used for this regard (34, 35). Sample size sufficiency for performing EFA was determined by KMO (Kaiser Meyer Olkin) and BTS (Bartlett’s Test of Sphericity) (36, 37).

### CFA

The factors extracted in the previous step were assessed using AMOS version 24. Before conducting CFA, outlier data were assessed by Mahalanobis. Then, data normality was checked using kurtosis and skewness. The goodness of fit indexes such as GFI (goodness of fit index), PNFI (parsimonious normed fit index), χ2/df (chi-square ratio to degree of freedom), PGFI (parsimony goodness of fit index), IFI (incremental fit index), RMSEA (root mean square error of approximation), PCFI (parsimony comparative fit index), and CFI (comparative fit index) were used to verify and confirm the final model (38–41). Based on resources, the standard value for each index is RMSEA < 0.08, PNFI > 0.5, GFI > 0.9, χ2/df < 5, PGFI > 0.5, IFI > 0.9, PCFI > 0.5, and CFI > 0.9 (38–41).

### Reliability

Three methods were used to measure scale reliability. Internal consistency was checked by two tests of Cronbach’s alpha coefficient (in SPSS software version 24) and McDonald Omega coefficient (in JASP software version 0.11.1.0) among 30 participants. Sources have recommended that a score ranging from 0.70 to 0.95 is good for internal reliability (42, 43). Also, in the test– retest, the intraclass correlation coefficient (ICC) was calculated. ICC was checked using SPSS software version 24, and an ICC of more than 0.80 is good. In this study, test-retest was performed on 30 participants and data were gathered twice (second time was gathered after 1 month).

## Results

### Demographic characteristics

The mean (± standard deviation) ages of participants in EFA and CFA were 32.19 (± 12.15) and 34.28 (± 13.68). Other demographic information was mentioned in **Table 1**.

**Table 1:** Frequency distribution of demographic characteristics.

Variables		EFA (n=399)		CFA (n= 557)	
		n	%	n	%
**Sex**	Male	174	43.6	281	50.4
	Female	225	56.4	276	49.6
**Occupation**	Housewife	35	8.8	74	13.3
	Employed	116	29.1	128	23
	University student	163	40.9	208	37.3
	Unemployed	6	1.5	14	2.5
	Self-employed	59	14.8	78	14
	laborer	5	1.3	18	3.2
	Retired	15	3.8	37	6.6
**Age group**	≤ 28	207	51.9	245	44
	29-38	68	17	121	21.7
	39-48	72	18	92	16.5
	49-58	40	10	63	11.3
	> 58	12	3	36	6.5
**Marital status**	Married	226	56.6	323	58
	Single	173	43.9	235	42.1
**Economic status**	Weak	46	11.5	68	12.2
	Medium	266	66.7	375	67.3
	Excellent	87	21.8	114	20.5
**Education level**	Elementary school	4	1	13	2.3
	Middle school	9	2.3	23	4.1
	High school	7	1.8	29	5.2
	Diploma	112	28.1	156	28
	Associate degree	56	14	84	15.1
	Bachelor degree	155	38.8	185	33.2
	Master’s degree or high degree	56	14	67	12

### Face and content validity

In face validity and content validity, 4 items and 3 items were modified (used appropriate and simple words.), respectively. Also, the score of S-CVI/Ave and CVR for SOSS were 0.982 and 0.921, respectively.

### EFA

**Figure 1:**
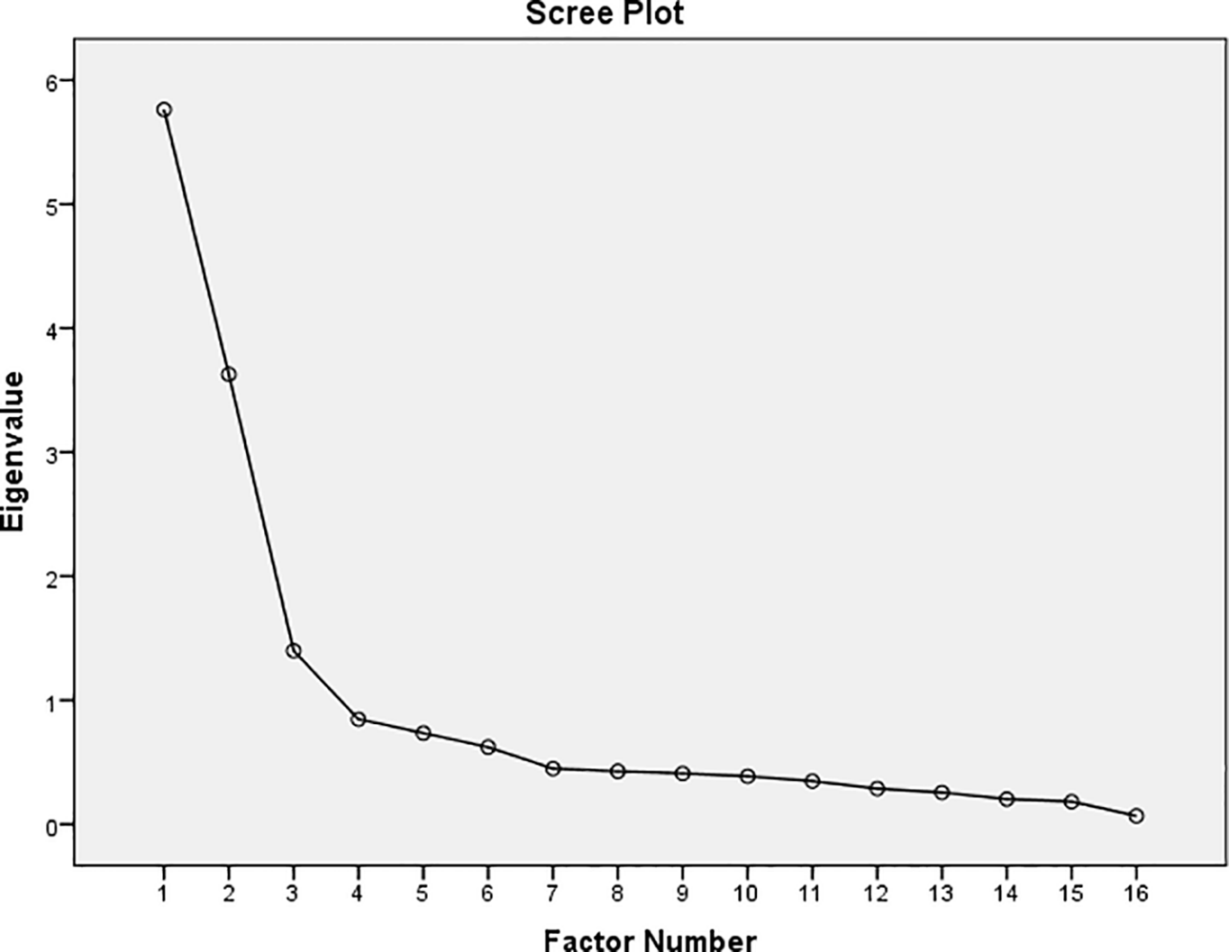
Scree plot of the factor analysis of the Persian short form of SOSS.

In this section, evaluation of sample size adequacy for performed EFA was done using KMO and BTS (KMO = .877, Bartlett’s test: p <.001, χ2 = 4215.937, df = 120). In EFA, three factors with eigenvalues above one were shown, and 60.60% variance of scale was explained by these factors (**Table 2**; **Figure 1**). In this section, in the EFA, one question (*I think people who commit suicide are Pathetic*) was eliminated due had factor loading less than 0.4 and also moved to an irrelevant factor (**Table 3**).

**Table 2:** The three-factor structure of the Persian short form of SOSS.

Total Variance Explained									
Component	Initial Eigenvalues			Extraction Sums of Squared Loadings			Rotation Sums of Squared Loadings		
	Total	% Of Variance	Cumulative %	Total	% Of Variance	Cumulative %	Total	% Of Variance	Cumulative %
1	5.762	36.014	36.014	3.526	22.034	22.034	3.612	22.576	22.576
2	3.626	22.665	58.680	5.172	32.325	54.360	3.433	21.458	44.034
3	1.398	8.740	67.419	.999	6.245	60.605	2.651	16.571	60.605
4	.847	5.291	72.710						
5	.734	4.589	77.299						
6	.621	3.884	81.183						
7	.448	2.798	83.982						
8	.427	2.668	86.649						
9	.409	2.556	89.205						
10	.386	2.414	91.619						
11	.347	2.170	93.789						
12	.287	1.796	95.585						
13	.256	1.599	97.185						
14	.203	1.266	98.450						
15	.181	1.134	99.584						
16	.067	.416	100.000						

Extraction Method: Maximum Likelihood.

**Table 3:** Rotated factor matrix of the Persian short form of SOSS.

Rotated Factor Matrix^a^			
Items	Factor		
	1	2	3
**N6**	**.772**	-.109	.217
**N7**	**.768**	-.114	.314
**N3**	**.760**	-.043	.171
**N5**	**.756**	.055	.259
**N2**	**.645**	-.028	.192
**N8**	**.484**	.249	.333
**N4**	**.454**	.294	.351
**N15**	-.007	**.973**	.057
**N16**	-.015	**.949**	.041
**N14**	.010	**.865**	.078
**N13**	-.023	**.777**	.065
**N10**	.288	-.004	**.776**
**N9**	.150	.207	**.718**
**N11**	.276	-.029	**.665**
**N12**	.326	.024	**.639**
**N1**	.363	.100	**.392**

Extraction Method: Maximum Likelihood.

Rotation Method: Varimax with Kaiser Normalization.

a Rotation converged in 5 iterations. Bold values show questions related each factor.

### CFA

**Figure 2:**
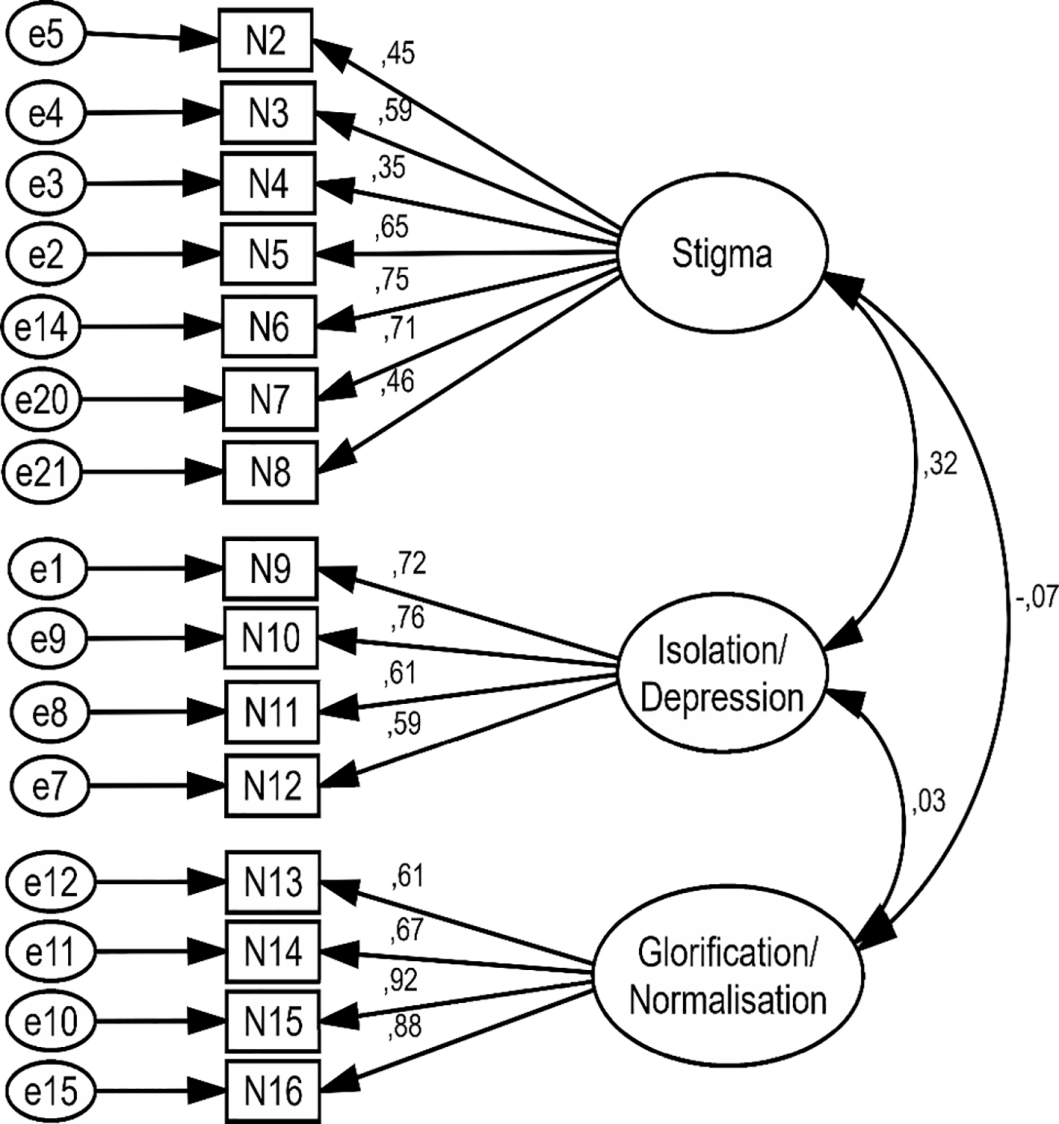
Standardized parameter estimates for the factor structure of the Persian short form of SOSS in first model.

**Figure 3:**
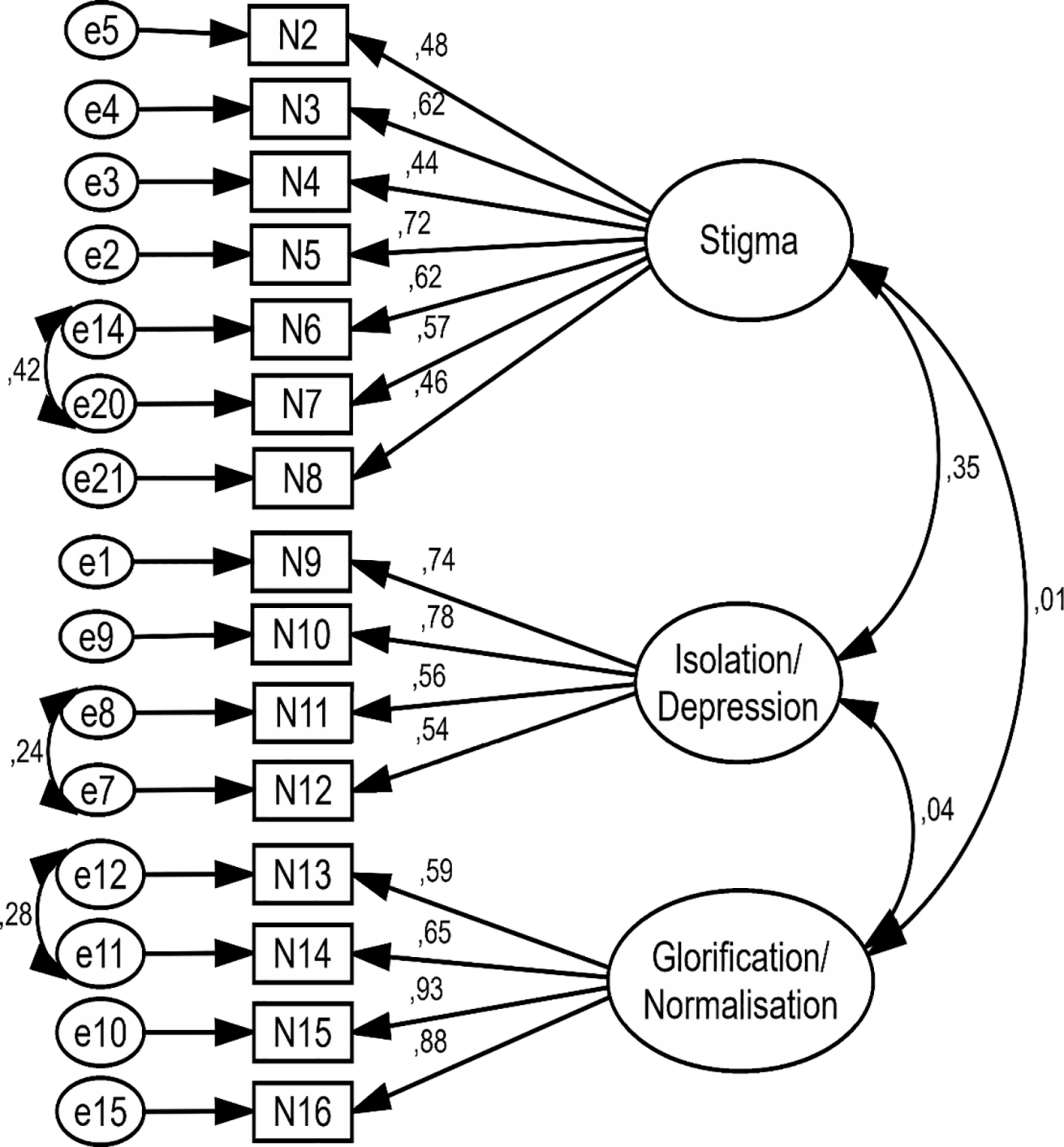
Standardized parameter estimates for the factor structure of the Persian short form of SOSS in second model.

The three factors extracted in the EFA stage, were evaluated in CFA. The final model of the Persian short form of SOSS was drawn and confirmed using AMOS version 24 software. In this model, Standardized parameter estimates for the factor structure of the Persian short form of SOSS was shown. In the confirmed model, the big circles represent the three subscales of SOSS and the rectangles represent the items related to each subscale. The two-way arrows between the large circles show the correlation between the subscales. One-way arrows from large circles to rectangles show which items load on which factor, and the values mentioned on each arrow indicate the standardized regression coefficient (or factor loading) of each item. The small arrows from the small circles (e) to the rectangles show the residual variance (error) (**Figures 2**, **3**).

In the first model, all items of SOSS had factor loading more than 0.4 without used any modification index (**Figure 2**). In the second model, three modification indexes were created between measurement error (e 14 to e20 in stigma, e7 to e8 in isolation/depression, and e11 to e12 in glorification/normalization). In second model, after created measurement error the factor loading of some items of SOSS were improved and all items of SOSS had factor loading more than 0.4 (**Table 4**; **Figure 3**).

**Table 4:** Factor loadings of the Persian short form of SOSS in EFA and CFA.

Factors	Items	Factor loadings	
		EFA	CFA
**Stigma**	1. Pathetic	0.392	*Deleted*
	2. Shallow	0.645	0.477
	3. Immoral	0.760	0.617
	4. An embarrassment	0.454	0.436
	5. Irresponsible	0.756	0.725
	6. Stupid	0.772	0.620
	7. Cowardly	0.768	0.565
	8. Vengeful	0.484	0.458
**Isolation/Depression**	9. Lonely	0.718	0.739
	10. Isolated	0.776	0.784
	11. Lost	0.665	0.555
	12. Disconnected	0.639	0.536
**Glorification/Normalization**	13. Strong	0.777	0.588
	14. Brave	0.865	0.653
	15. Noble	0.973	0.926
	16. Dedicated	0.949	0.881

In the first model, before used any modification index the goodness-of-fit indexes (such as IFI= .860, RMSEA= .091, CFI= .859, GFI= .884) were not appropriated (**Table 5**). But, after used the three modification indexes between measurement error, the goodness-of-fit indexes (such as IFI= .903, RMSEA= .077, CFI= .902, GFI= .915) were improved and got acceptable values (**Table 5**). Finally, based on the goodness-of-fit indexes results, the Persian short form of SOSS with 15 items and three factors of glorification/normalization (4 items), stigma (7 items), and isolation/depression (4 items) was approved (**Table 5**; **Figure 3**). Also, the score range for each subscale is between 1–5. The final Persian short form of SOSS was uploaded as **Supplementary Material** (**Appendix 1**).

**Table 5:** The model fit indicators of the Persian short form of SOSS.

Goodness of fit indices	Confirmatory factor analysis (Before modification index)	Confirmatory factor analysis (After modification index)	Acceptable value
**X^2^**	489.752	364.443	–
**df**	87	84	–
**X^2^/df**	5.629	4.339	< 5
**p-value**	0.000	0.000	p > 0.05
**CFI**	0.859	0.902	> 0.9
**RMSEA**	0.091	0.077	<0.08
**GFI**	0.884	0.915	> 0.9
**IFI**	0.860	0.903	> 0.9
**PNFI**	0.692	0.702	> 0.5
**PGFI**	0.641	0.640	> 0.5
**PCFI**	0.712	0.722	> 0.5

### Reliability assessment

The McDonald Omega coefficient, Cronbach’s alpha coefficient, and ICC for the total items of the SOSS were 0.841, 0.834, and 0.881, respectively. The reliability results for each factor have been listed in **Table 6**.

**Table 6:** Descriptive statistics of the Persian short form of SOSS.

Factors	Item	Range	Internal consistency (n=30 participants)		Test-retest (n=30 participants)			
			Cronbach’s alpha coefficients	McDonald Omega coefficient	Intraclass Correlation Coefficient (ICC)	95% Confidence Interval		P-value
						Lower Bound	Upper Bound	
**Stigma**	7	7–35	0.875	0.877	0.804	0.589	0.907	<0.001
**Isolation/Depression**	4	4–20	0.760	0.783	0.964	0.925	0.983	<0.001
**Glorification/Normalization**	4	4–20	0.937	0.939	0.985	0.968	0.993	<0.001
**Total SOSS**	15	15–75	0.834	0.841	0.881	0.750	0.943	<0.001

## Discussion

In this study, we examined the validity and reliability of the Persian short form of SOSS in the general population of Iran. The original version of the short form of the SOSS consisted of 16 items; however, after evaluating the psychometric properties in this study, one question was removed from the Persian short form of SOSS and the modified version was confirmed with 15 items and three factors. Therefore, with the approval of the psychometric properties of the Persian short form of SOSS in general population, the SOSS can be used to measure suicide stigma in target populations.

Based on the EFA results in our study, three factors with eigenvalue values more than one were able to explain more than two -thirds of the variance. Based on the EFA, all the factor loading values were greater than 0.4, and only one question (I think people who commit suicide are pathetic) was eliminated due to the move to the irrelevant factor and had factor loading less than 0.4. This item has also been removed in other versions of SOSS in other countries. For example, in the Chinese version of the questionnaire (44), four items (pathetic, irresponsible, disconnected, cowardly), and in the Bangladeshi version (10), three items (pathetic, an embarrassment, shallow) were removed due to the low factor loading.

In our study, items “embarrassment” and “vengeful” from the stigma subscale had marginal factor loadings of 0.454 and 0.484, respectively. These factor loadings show that these two items were less related to stigma structure than the other items. In the case of “embarrassment”, it seems that the possible reasons are cultural and perceptional. In fact, in our society, people can easily attribute the adjective “embarrassment” to themselves or others without the intention of labeling or a negative attitude, and it is a common attribute. When the “embarrassment” is attributed to people, they don’t feel much stigma. In addition, in the perception of all people, embarrassment has less negative content and stigma compared to, for example, being stupid (factor loading= 0.772). Therefore, these two reasons can somehow justify the lower correlation between “embarrassment” item and the stigma subscale. In the case of the “vengeful” attribute, not specifying the direction of revenge can cause ambiguity in participants’ perceptions of this item. In fact, it is not clear whether suicidal people intend to take revenge on themselves or whether they intend to take revenge on others. This equivocality and different perceptions can be the origin of the marginal factor loading of this item. As a result, future studies should pay more detailed attention to these issues.

In the psychometric evaluation of short form of SOSS in different countries including China, Bangladesh (10, 44), and in our study in Iran, the first item, “pathetic”, had a low factor loading, and as a result, this item was deleted. In analyzing the reason for removing this item, our argument is, first these countries are located in different geographical locations, secondly, they have a relatively different economic status, and thirdly, they are completely different in terms of religion, values, norms, cultural issues and customs. Therefore, the reason for this similarity between these four studies regarding the deletion of the first item cannot be attributed to geographical, economic, cultural and social factors. In our opinion, the nature of the equivocality of the word “pathetic” is one of the possible causes of this problem, which causes disruption in the translation process and, as a result, the interpretation of this item by the participants.

In fact, in the Cambridge dictionary, this word has two categories of meanings in two directions: 1. meaning with a positive theme: “causing feelings of sadness, sympathy, especially because a person or an animal is suffering” and 2. Meaning with negative theme: unsuccessful or showing no ability, effort, or bravery, so that people feel no respect (45). The first meaning is not considered as a label and stigma and it can be said that it is synonymous with emotional (46). As a result, if the participants had this interpretation of this item, it seems logical to answer this item differently than other items of the stigma subscale. Therefore, the low factor loading of this item seems reasonable. In the Chinese study, the difference in the intensity of emotionality was mentioned as a reason (44). But apparently, the purpose of the SOSS designer is the second meaning, which is synonymous with pitiful and insufficient. Therefore, it is suggested that future studies take into account the translation intended by the designer to psychometrically analyze this questionnaire.

Although one question was eliminated in the Persian short form of SOSS but all factors of the main questionnaire were confirmed, which is largely in line with other studies (10, 44). For example, in a study conducted on Jordanian students, the short Arabic version of the SOSS questionnaire was confirmed with three factors and 16 questions (3). In Chinese version of SOSS, 4 questions were eliminated, and final version was confirmed with 12 questions and 3 factors of Glorification/Normalization (4 items), Stigma (5 items)and Isolation/Depression (3 items) (44). In a study conducted among Bangladeshi students, three questions were eliminated and the questionnaire was finally confirmed with 13 questions and three factors of Glorification/Normalization (4 items), Stigma (5 items) and Isolation/Depression (4 items) (10). This difference may be due to the sociocultural differences of countries that affect the state of suicide stigma (47, 48).

In this study, McDonald Omega coefficient, Cronbach’s alpha coefficient, and ICC were used to measure the reliability of the tool, and were 0.841, 0.834, and 0.881, respectively. Cronbach’s alpha coefficient values were also appropriate for three factors of Glorification/Normalization (α=0.937), Stigma (α=0.875), and Isolation/Depression (α=0.760). The results of this study were consistent with the results of several studies in other countries (3, 10, 15, 44).

A study among Chinese students showed that Cronbach’s alpha coefficient was 0.72, 0.85, and 0.77, respectively, for Stigma, Isolation/Depression, and Glorification/Normalization, which confirmed the internal consistency reliability of the SOSS (44). In another study in Australia, the three factors of SOSS had an acceptable Cronbach’s alpha coefficient (> 0.7) (15). In another study in Jordan, Cronbach’s alpha coefficient for the short Arabic version of SOSS was acceptable and for three factors of Stigma, Isolation/Depression, and Glorification/Normalization calculated 0.81, 0.71, and 0.68, respectively (3). A study in Bangladesh assessed the reliability of the short version of the SOSS on university students and showed that Cronbach’s alpha coefficient for factors of stigma, isolation/depression, and glorification/normalization were 0.76, 0.88, and 0.68 (10).

Given that the cultural and socioeconomic status of countries affect the viewpoints of the community and suicide stigma (49), the status of suicide stigma in each country needs to be examined and necessary preventive measures should be designed and implemented. Therefore, with the approval of the Persian version of the short form of the SOSS in this study, this localized questionnaire can be used to measure the status of suicide stigma in different groups and regions of Iran to determine the status of suicide stigma and to design and implement appropriate preventive programs if needed.

SOSS-15 can be used as a useful screening tool for suicide prevention in public health or an evaluation tool for clinical procedures. Needs assessment is inseparable and considered as the most important stage of any educational process. Therefore, educational interventions aimed at reducing the suicide stigma can be more targeted, effective and efficient when the prevalence of suicide stigma in the society is first determined. As a result, SOSS can be used for screening people in terms of suicide stigma, helping researchers to design and implement targeted research interventions, and a suitable tool for health care professionals and health policy makers.

### Strengths and limitations

The strengths of the present study include the large sample size and the study of different age groups and social classes. Although different groups have been included, but one of the limitations of this study was that most of the participants were female and university students. So, it is recommended that future studies be conducted in such a way that the demographic characteristics of the sample (such as gender composition) are more consistent with the general population. Due to not checking criterion validity in this study, it is suggested that future studies be conducted in this regard and check the sensitivity, specificity and optimal cut-off points for questionnaire.

## Conclusion

In this study, the Persian short form of SOSS was approved with 15 items and 3 factors (stigma with 7 questions, isolation/depression with 4 questions, and glorification/normalization with 4 questions), and this scale is an appropriate instrument for measuring suicide stigma among general population. Therefore, given the importance of localization of SOSS, it is recommended to use this questionnaire to determine the status of suicide stigma in different groups and regions of Iran. Also, after determining the suicide stigma rate in different regions of Iran, effective national interventions can be design and implement by researchers, health care providers, and policy makers.

## Data availability statement

The datasets presented in this study can be found in online repositories. The names of the repository/repositories and accession number(s) can be found in the article/**Supplementary Material**.

## Ethics statement

This study is based on a research project approved by Ethics Committee of Gonabad University of Medical Sciences with the code of ethics IR.GMU.REC.1401.090. All procedures performed in this study were in accordance with the ethical standards of the institutional and/or national research committee and with the 1964 Helsinki declaration and its later amendments or comparable. The studies were conducted in accordance with the local legislation and institutional requirements. The participants provided their written informed consent to participate in this study.

## Author contributions

AJ: Writing – review & editing, Writing – original draft, Validation, Software, Project administration, Methodology, Investigation, Formal analysis, Data curation, Conceptualization. MM: Writing – review & editing, Writing – original draft, Validation, Methodology, Investigation, Conceptualization. AM: Writing – review & editing, Writing – original draft, Software, Methodology, Investigation, Conceptualization. FR: Writing – review & editing, Writing – original draft, Investigation, Conceptualization. FN: Writing – review & editing, Writing – original draft, Investigation, Conceptualization. MN: Writing – review & editing, Writing – original draft, Validation, Supervision, Methodology, Investigation, Conceptualization.
