# Supplementary material for: Psychometric properties of the Persian short form of the Stigma of Suicide Scale
Source: Front Psychiatry. 2024 Jul 26;15:1394237. doi: 10.3389/fpsyt.2024.1394237 (PMC11310839; doi:10.3389/fpsyt.2024.1394237)
Supplement: Supplementary file 1 [file Table_1.docx]

**Appendix 1:** The final Persian short form of Stigma of Suicide Scale (SOSS) with 15 items and 3 factors

| **نسخه نهایی فرم کوتاه پرسشنامه فارسی استیگمای خودکشی (15 سوال)** | **کاملا مخالفم** | **مخالفم** | **نه مخالف نه موافق** | **موافقم** | **کاملا موافقم** |
| --- | --- | --- | --- | --- | --- |
| 1. به نظر من کسانی که خودکشی می کنند افرادی سطحی نگری هستند | **Stigma** | | | | |
| 1. کسانی که خودکشی می کنند احساس مسئولیت اخلاقی ندارند |  |  |  |  |  |
| 1. به نظر من افرادی که خودکشی می کنند خجالتی هستند |  |  |  |  |  |
| 1. کسانی که خودکشی می کنند افراد بی مسئولیتی هستند |  |  |  |  |  |
| 1. خودکشی نشانه حماقت است |  |  |  |  |  |
| 1. به نظر من خودکشی کار افراد ضعیف النفس است |  |  |  |  |  |
| 1. افرادی که خودکشی می کنند انتقام جو هستند |  |  |  |  |  |
| 1. به نظر من کسانی که خودکشی می کنند افراد تنها و بی کسی هستند | **Isolation/ Depression** | | | | |
| 1. افرادی که خودکشی می کنند منزوی هستند |  |  |  |  |  |
| 1. به نظر من افرادی که خودکشی می کنند شکست خورده هستند |  |  |  |  |  |
| 1. به نظر من کسانی که خودکشی می کنند ارتباطشان با سایر افراد قطع شده است |  |  |  |  |  |
| 1. به نظر من افرادی که خودکشی می کنند قوی هستند | **Glorification/ Normalisation** | | | | |
| 1. خودکشی در افرادی که آن را انجام می دهند نشانه شجاعت است |  |  |  |  |  |
| 1. به نظر من افرادی که خودکشی می کنند سخاوتمند هستند |  |  |  |  |  |
| 1. به نظر من افرادی که خودکشی می کنند فداکار هستند |  |  |  |  |  |
